# Recent Advance in Heterocyclic Organozinc and Organomanganese Compounds; Direct Synthetic Routes and Application in Organic Synthesis

**DOI:** 10.3390/molecules15118006

**Published:** 2010-11-08

**Authors:** Seung-Hoi Kim, Reuben D. Rieke

**Affiliations:** 1 Department of Chemistry, Dankook University; 29 Anseo Cheonan Chungnam, 330-714, Korea; E-Mail: kimsemail@dankook.ac.kr (S-H.K.); Tel.: 82-41-550-1816; 2 Rieke Metals, Inc.; 1001 Kingbird Rd. Lincoln, NE 68512, USA

**Keywords:** highly active metals, organozinc, organomanganese, heterocyclic compounds

## Abstract

A practical synthetic route for the preparation of 2-pyridyl and 3-pyridyl derivatives has been accomplished by utilizing a simple coupling reaction of stable 2-pyridylzinc bromides and 3-pyridylzinc bromides. The organozincs used in this study were easily prepared via the direct insertion of active zinc into the corresponding bromopyridines. The subsequent coupling reactions with a variety of different electrophiles have afforded the corresponding coupling products. Using highly active manganese, a variety of Grignard-type organomanganese reagents have been obtained. The subsequent coupling reactions of the resulting organomanganese reagents with several electrophiles have also been accomplished under mild conditions.

## 1. Introduction

In 1972, Rieke and co-workers reported a general approach for preparing highly active metal powders (called Rieke metals) by reducing metal salts in ethereal or hydrocarbon solvents using alkali metals as reducing reagents [1]. A second general approach for the preparation of Rieke metals involves using an alkali metal in conjunction with an electron carrier, such as naphthalene or biphenyls. Using the Rieke method, many metals on periodic table, most notably copper, zinc, magnesium, calcium, indium, nickel, cobalt, iron, and barium, have been activated and used in the synthesis of many organic compounds. [2,3]. In addition to these metals, highly active manganese has also been prepared using the general Rieke method [4]. Using this highly active zinc and manganese, a variety of organozinc and organomanganese reagents have been prepared and applied in organic synthesis. Remarkably, direct synthesis of Grignard-type of heteroarylzinc and manganese reagents has been successfully achieved. It is of importance that the resulting organometallic reagents also showed excellent reactivity to give cross-coupling products under mild conditions. Among many heteroaromatic reagents prepared by the Rieke method, pyridyl- and thienyl-metallic reagents are critically reviewed in this report.

Heterocyclic compounds which contain a pyridine function have been frequently found in natural products that have biological activity, especially in pharmaceutical, agrochemical and medicinal chemistry [5,6,7]. And also, a number of pyridine derivatives have been used in material chemistry [8,9]. Consequently, new practical synthetic approaches for introducing a pyridine ring into complex organic molecules are of high value. To this end, preparations of pyridyl derivatives are mostly performed by transition metal catalyzed cross-coupling reactions of pyridylmetallic reagents. However, the preparation of electron-deficient pyridyl organometallic reagents has been a challenging subject, mainly because of some difficulties such as instability and formation of by-products [10].

Most of the 2-pyridyl derivatives have been prepared using the Suzuki [11,12,13,14], Stille [15,16,17], Grignard [18,19,20], and Negishi [21,22,23,24] coupling reactions in the presence of a transition metal catalyst. Among these, the Suzuki coupling reaction is the most intensively studied and a very extensive body of work has been developed in this area [25]. Recently, several outstanding studies on the direct arylation of pyridine have been reported to avoid the classical inevitable difficulties. For example, Rh(I) [26] and Au(I)-catalyzed [27] arylation of pyridines, Pd-catalyzed arylation of pyridine N-oxide with unactivated arenes [28] and haloarenes [29] have all been developed. The direct arylation of pyridine N-oxide by Grignard reagents was also reported [30].

Even though there are many examples of the preparation of 2-pyridylmetallic halides via the reaction of halopyridines, a limited number of studies have been reported on the preparation of 3-pyridylmetallic halides. 3-Pyridylmagnesium [31,32,33], 3-pyridylzinc [34], 3–pyridylindium [35,36,37] halides and Suzuki reagents [38,39,40,41,42,43] are the most widely used reagents for the preparation of pyridine-containing compounds. Lithiation of 3-halopyridine followed by transmetallation with appropriate metals (Mg, Zn, In) afforded the corresponding 3-pyridylmetallic halides. However, this route has limitations such as the need for cryogenic conditions, several side reactions and limited functional group tolerance [44]. Very few studies have been reported on the direct synthesis of 3-pyridylmetallic halide reagents. Most of these reports included the treatment of 3-iodo or 3-bromopyridine with highly active metals [45,46,47].

Along with other Rieke metals, Rieke manganese can be also easily prepared by reduction of anhydrous manganese halides using lithium in the presence of a catalytic amount of naphthalene in THF at room temperature. The resulting active manganese is one of the most reactive metals and readily undergoes oxidative addition with some of the unreactive halides. Alkyl- and aryl manganese bromides can be easily generated by direct oxidative addition of highly active manganese to alkyl and aryl bromides. These organomanganese reagents have been found to undergo cross-coupling reactions with a variety of electrophiles including acid chlorides to give the corresponding ketones in good yields. Based upon these results, several different types of organomanganese reagents have been prepared and utilized in the field of organic chemistry. One of the interesting results obtained using the highly active manganese is 3-thienylmanganese halide.

3-Substituted thiophene derivatives have been utilized for the preparation of useful compounds in both materials and pharmaceutical science. For instance, poly(3-alkyl thiophene) films have good processability potential [48,49,50,51,52] and have been found to be stable for extended periods under a variety of conditions. More interestingly, 3-substituted thiophene derivatives have been found to be topical carbonic anhydrase inhibitors [53]. 

To date, the most widely used methods for the preparation of 3-substituted thiophene derivatives are the coupling reactions of 3-thienyl organometallic reagents with electrophiles. The intermediates used in these reactions are generally obtained via either a metal-halogen exchange reaction of 3-bromo-thiophene with *n*-butyllithium [54] or the metathesis of 3-lithiothiophene with different metal halides [55,56]. However, the utility of these reactions is limited owing to the lack of regiospecificity as well as observed decomposition of the thiophene ring at room temperature [57]. 

Direct preparation of 3-thienylzinc bromide using a simple electrochemical method was reported [58]. Also, the direct oxidative addition of Rieke zinc (Zn*) and Rieke magnesium (Mg*) to 3-iodothiophene were completed under mild conditions in high yields and the coupling reactions of the resulting 3-thienylzinc and 3-thienylmagnesium iodides with electrophiles were performed [59]. Surprisingly, use of Rieke manganese provides 3-thienylmanganese bromide from 3-bromothiophene. The coupling reaction products are also obtained when the resulting 3-thienylmanganese bromide is reacted with various electrophiles [60]. It is interesting to note that this methodology can be applied to 3,4-dibromothiophene in the preparation of symmetrical and/or unsymmetrical 3,4-disubstituted thiophene derivatives.

## 2. Results and Discussion

### 2.1. Heteroarylzinc reagents

In general, the preparation of 2-pyridyl organometallics is mostly performed by lithiation of 2-halo-pyridine under cryogenic conditions followed by transmetallation with an appropriate metal halide. As mentioned above, this procedure imposes some limitations on the use of the 2-pyridyl organometallics. Treatment of readily available 2-bromopyridine with the active zinc gave the corresponding organozinc reagent. The oxidative addition of the active zinc to carbon-bromine bond was completed in an hour at refluxing temperature to give rise to the corresponding 2-pyridylzinc bromide (**P1**). 

In order to investigate the reactivity of the 2-pyridylzinc bromide, it was treated with benzoyl chlorides. As summarized in Table 1, the coupling ketone products were obtained in moderate yields. It should be emphasized that the coupling reaction with acid chlorides was carried out in the absence of any transition metal catalyst under mild conditions. Generally, a copper catalyst is widely used for the coupling reactions of organozinc reagents [61]. Halobenzoyl chlorides were easily coupled with 2-pyridylzinc bromide (**P1**) at rt to give the corresponding ketones (**1a**, **1b**, **1c**, **1d** and **1e**, Table 1) in moderate yields. Both benzoyl chlorides containing an electron-withdrawing group (CN and NO_2_) and an electron-donating group (Me and MeO) also successfully afforded the corresponding ketones(**1f**, **1g**, **1h**, and **1i**, Table 1). Even with nitrobenzoyl chloride, ketone (**1j**, Table 1) was obtained in moderate yield. 

**Table 1:** Coupling reaction with benzoyl chlorides.^ a^

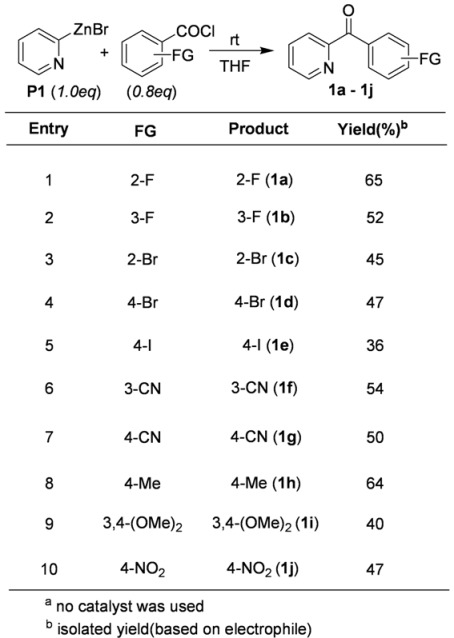

More results obtained from the catalyst-free coupling reactions are shown in Table 2. Treatment of **P1** with chloronicotinoyl chlorides and alkyl carbonyl chlorides provided the corresponding ketones. This result is particularly significant considering the fact that Fridel-Craft acylation can be accomplished on the pyridine ring.

Together with these results, the Pd-catalyzed C-C bond forming reaction of **P1** was also explored. Even though 2-pyridylaryl derivatives were successfully prepared via the aforementioned direct arylation methods, relatively harsh conditions (excess amount of reactant, high temperature, protection/deprotection step and addition of additives) were required in these studies. 

Prior to the Pd-catalyzed coupling reaction with a variety of arylhalides, an effort was executed to find out any effect of substitutents on the cross-coupling reactions. In general, good yields (entries 1, 3, and 4, Table 1) were obtained from using 2-pyridylzinc bromide (**P1**), 4-methyl-2-pyridylzinc bromide (**P3**), and 5-methyl-2-pyridylzinc bromide (**P4**).

**Table 2:** Coupling reaction with acid chlorides.^ a^

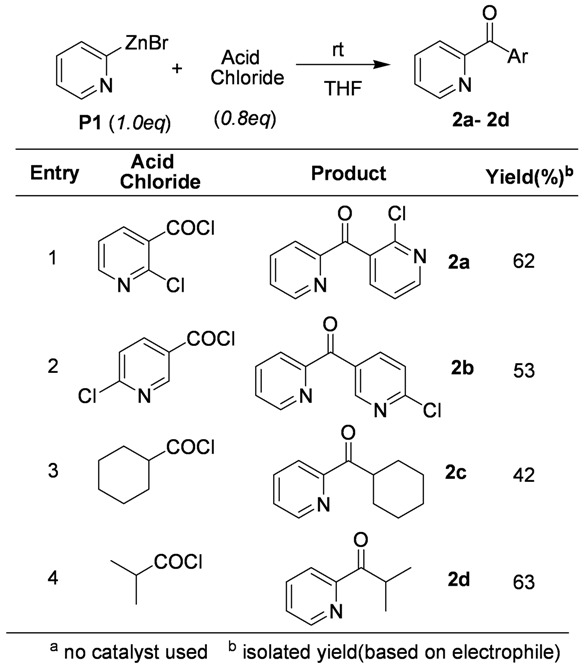

3-Methyl-2-pyridylzinc bromide (**P2**), 6-methyl-2-pyridylzinc bromide (**P5**), and 6-methoxy-2-pyridylzinc bromide (**P6**) resulted in moderate yields (entries 2, 5, and 6, Table 3).

**Table 3:** Study of substitutent effect.

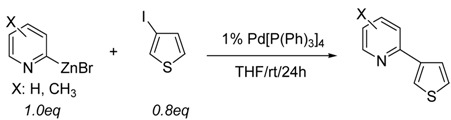
Entry	X	Product, X	Yield(%)^a^
1	H(**P1**)	H (**3a**)	85
2	3-CH_3_(**P2**)	3-CH_3 _(**3b**)	58
3	4-CH_3_(**P3**)	4-CH_3 _(**3c**)	77
4	5-CH_3_(**P4**)	5-CH_3 _(**3d**)	79
5	6-CH_3_(**P5**)	6-CH_3 _(**3e**)	57
6	6-OCH_3_(**P6**)	6-OCH_3 _(**3f**)	54

^a^ isolated yield (based on 3-iodothiophene)

Additional studies have been performed to investigate the steric effect on cross-coupling reactions using 2-pyridylzinc bromides (**P2** and **P4**). As shown in Table 4, the effects of steric hindrance (76% *vs.* 89%, 51% *vs.* 84% isolated yield) were clearly observed from the coupling reactions with 5-bromofuran-2-carboxylic acid ethyl ester and 5-bromothiophene-2-carboxylic acid ethyl ester (entries 1, 2 and 3, 4, Table 4), respectively. The results demonstrate that the steric bulk around the reaction site reduces the coupling ability of the corresponding organozinc reagents. 

**Table 4:** Steric effect on cross-coupling reaction.

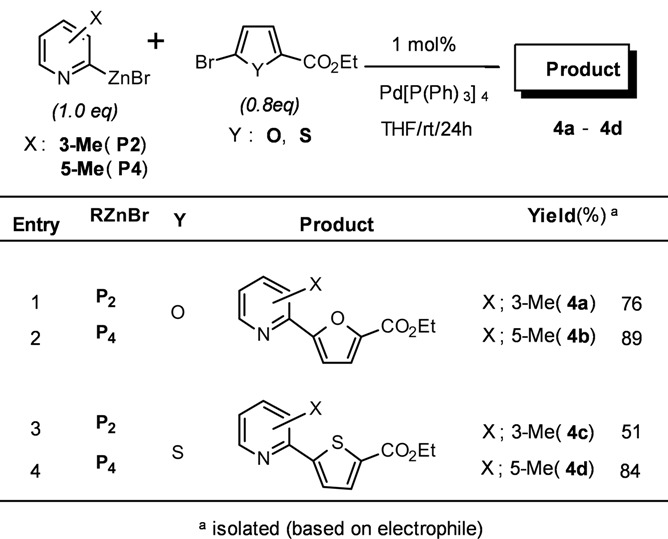

With the preliminary results, this methodology was expanded to coupling reactions with a variety of haloaromatic compounds. The results are described in Table 5. Interestingly, the mild conditions worked well allowing the coupling reactions of 2-pyridylzinc bromide (**P1**) to go to completion. As shown in Table 5, several different types of functionalized aryl halides and heteroaryl halides were coupled with **P1** in the presence of 1 mol% of Pd[P(Ph)_3_]_4_ at room temperature in THF. 

More interesting materials were prepared by the coupling reaction of various 2-pyridylzinc bromides with halo heterocyclic derivatives and the results are summarized in Table 6. A selective C-C bond forming occurred in the reactions with 2-bromo-3-hexyl-5-iodothiophene and 2-bromo-5-chlorothiophene. A slightly longer reaction time was required to complete the coupling reaction with 2-bromothiazole and 2-bromoquinoline with 4-methyl-2-pyridylzinc bromide (entries 2 and 3, Table 6). Significantly, another selective C-C bond forming was achieved from the coupling reaction with symmetrically substituted thiophene, 2,5-dibromothiophene (entry 5, Table 6). The resulting product, **6e**, could be used for further application. Even though a little bit different reaction conditions (Pd-II catalyst and refluxing temperature) was applied to carry out the coupling reactions with dibromothiophenes, symmetrically disubstituted thiophene derivatives (**6g** and **6h**) were easily prepared by its 2-fold reaction (entries 7 and 8, Table 6). These types of linear oligomers are important materials for optoelectronic device applications [62].

Bipyridines are very important moiety for natural product as well as other material chemistry. For example, caerulomycins and collismycins contain pyridine unit as a key material [63]. Therefore, it is worth to demonstrate to make these compounds by utilizing 2-pyridylzinc bromides used in this study. 

**Table 5:** Pd-catalyzed coupling of **P1** with arylhalide.

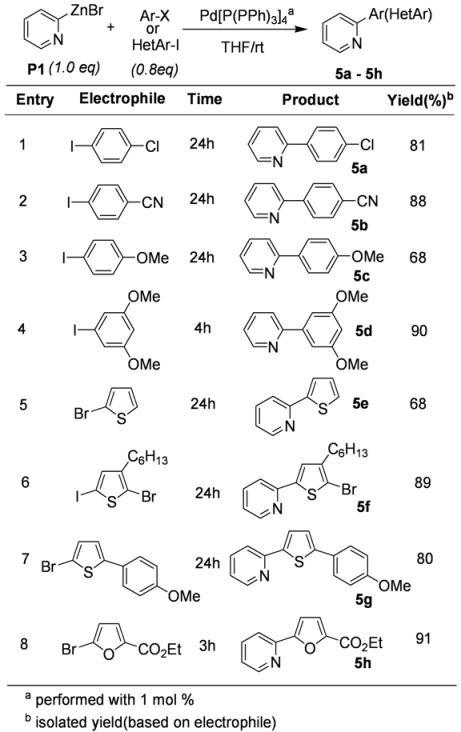

**Scheme 1:**
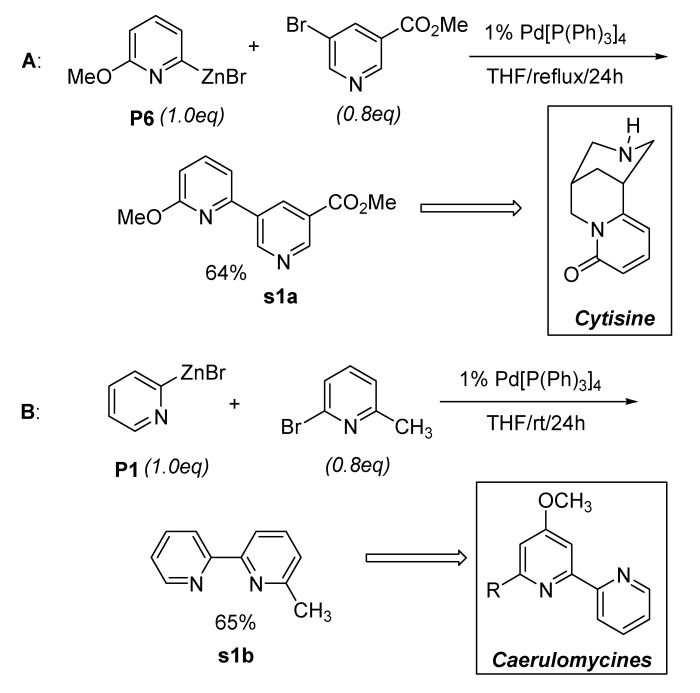
Preparation of intermediates.

As described in Table 7, not only symmetrical 2,2’-bipyridine (**7a**) but several different types of unsymmetrical 2,2’-bipyridines **7b-7h** were prepared in moderate yields. The preparation of bipyridines using readily available 2-pyridylzinc bromides **P1** ~ **P6** could be a very practical approach because considerable effort has been directed toward the preparation of unsymmetrical 2,2’-bipyridines. Scheme 1 shows two examples. 2,3-bipyridine (**s1a**) was prepared by the coupling reaction of **P6** with in the presence of Pd(PPh_3_)_4_ in THF at refluxing temperature affording the coupling product in 64% isolated yield (route **A**, Scheme 1). Under the similar conditions, 2,2’-bipyridine (**s1b**) was formed in moderate yield by Pd(0)-catalyzed cross-coupling reaction of **P1** (route **B**, Scheme 1). As described in Scheme 1, further applications of **s1a** and **s1b** could result in the formation of interesting natural products.

**Table 6:** More coupling reactions of 2-pyridylzincs with heteroaryl halides.

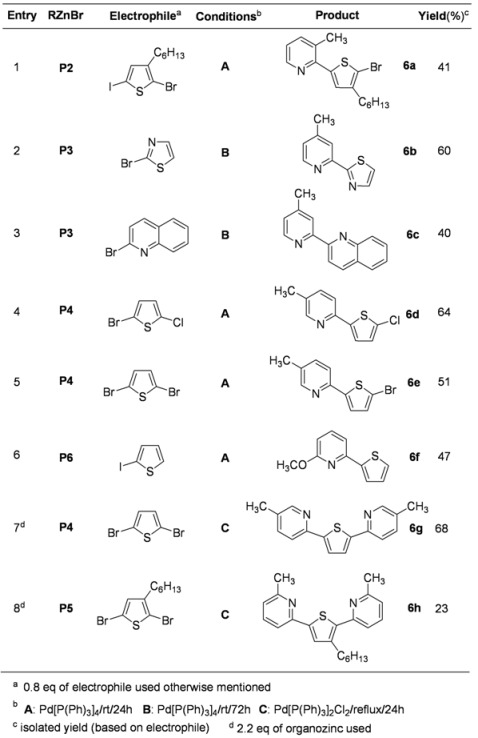

**Table 7:** Preparation of 2,2’-bipyridines^a^.

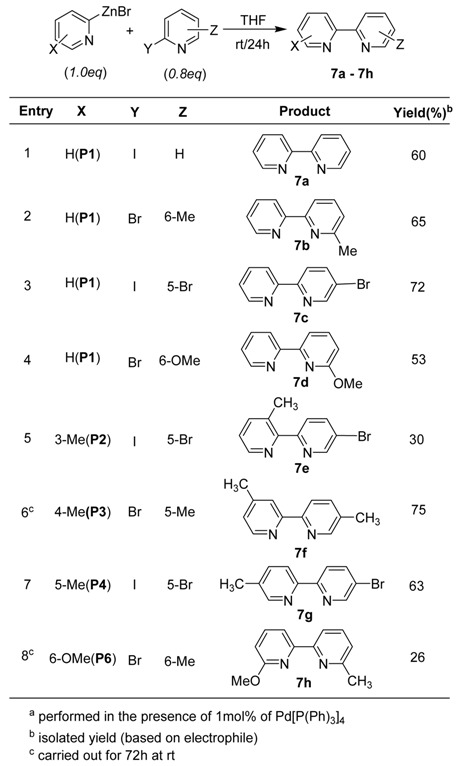

Most of the electrophiles used in the transition metal-catalyzed cross-coupling reactions of 2-pyridylmetallics contain functional groups that are relatively non-reactive toward organometallics, such as ester, ketone, nitrile, halogen, and ether. For the preparation of a variety of 2-pyridyl derivatives, highly functionalized electrophiles are necessary as the coupling partner in the reactions. To this end, haloaromatic amines, phenols and alcohols are reasonable coupling reactant candidates. By utilizing this strategy, 2-substituted aminophenyl and hydroxyphenyl pyridines have been successfully prepared under mild conditions. Since Pd(II)-catalysts along with an appropriate ligand have been used in the coupling reactions of organozinc reagents with haloaromatic amines and alcohols [64], it seemed reasonable to try these conditions. The coupling reactions worked well with 2-pyridylzinc bromide (**1a**) and the results are summarized in Table 8.

Methyl substituted 2-pyridylzinc bromide (**P3**) with 4-iodoaniline and **P1** with 4-bromoaniline resulted in relatively low yields (entries 2, 3, Table 8). However, a significantly improved yield was obtained by a simple change in reaction temperature (entry 4, Table 8). An elevated reaction temperature also worked well for the reaction of **P3** with 3-iodoaniline leading **8c** in 85% isolated yield (entry 5, Table 8). As described in the previous report [61,62,63,64], the extra ligand (SPhos) was critical for the completion of the coupling reaction.

**Table 8:** Coupling reaction with haloaromatic amines.

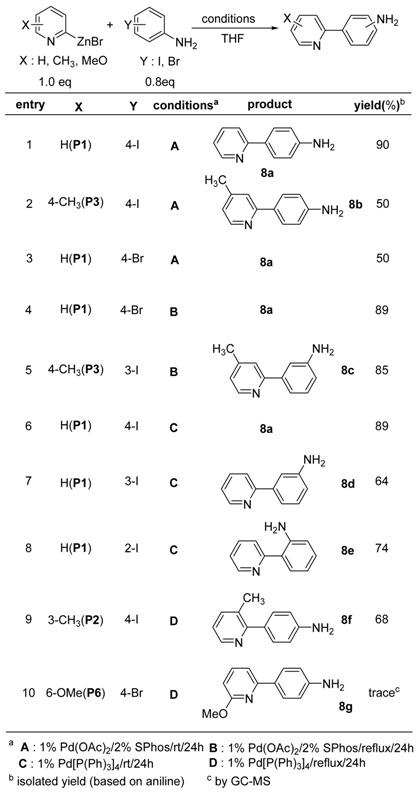

Even though similar conditions as in the previous works [64,65,66,67] were used, it should be emphasized that a more practical procedure, especially for the large scale synthesis, has been demonstrated in this study. For example, the organozinc solution was added into the flask containing Pd(II)-catalyst, ligand (SPhos) and electrophile at a steady-stream rate at rt. According to Knochel’s report, a very slow addition of organozinc reagent into the reaction flask was crucial in order to obtain high yields [68].

As mentioned above, the extra ligand (SPhos) was necessary when using the Pd(II)-catalysts for the coupling reactions. From an economic point of view as well as ease of work-up, a ligand-free reaction conditions would be highly beneficial. Thus the SPhos-free Pd-catalyzed coupling reactions of 2-pyridylzinc bromides with haloanilines were performed by employing a Pd(0)-catalyst and the results are summarized in Table 8. Significantly, the Pd(0)-catalyzed coupling reactions were not affected by the presence of acidic protons (NH_2_) [69]. 

The reaction of **P1** with 4-iodoaniline in the presence of 1 mol% Pd[P(Ph)_3_]_4_ provided 2-(4-amino-phenyl)pyridine (**8a**) with a compatible result (entry 6, Table 8). Unfortunately, no satisfactory coupling reaction occurred with 4-bromoaniline using the Pd(0)-catalyst (entry 10, Table 8). With the results obtained from the coupling reactions with haloaromatic amines, it can be concluded that Pd(0)-catalyzed reaction of 2-pyridylzinc bromides works effectively with iodoaromatic amines and also the relatively more reactive bromoaromatic amines.

Another interesting reaction of 2-pyridylzinc bromides would be the coupling reaction with phenols or alcohols, which also have an acidic proton. As shown in Table 9, 4-iodophenol and 3-iodophenol were coupled with **P1** affording the corresponding hydroxyphenylpyridine products **9a** and **9b** in excellent yields (entries 1 and 2, Table 9). A slightly disappointing result (25%) was obtained from 2-iodophenol (entry 3, Table 9). The reason for this is not clear, but it is presumably because the coupling position was next to the hydroxy group. A similar outcome has also been reported in another study [65]. In the case of bromophenolic alcohols, no coupling reaction took place with the Pd(0)-catalyst. Instead, the Pd(II)-catalyst was more efficient for the coupling reaction. Unlike the reactions with bromophenols, it is of interest that the coupling products (**9f** and **9g**) of **P5** and **P1** were efficiently achieved from the Pd(0)-catalyzed reactions with 4-bromobenzyl alcohol and 3-bromo-5-methoxybenzyl alcohol (entries 7 and 8, Table 9), respectively. 

**Scheme 2:**
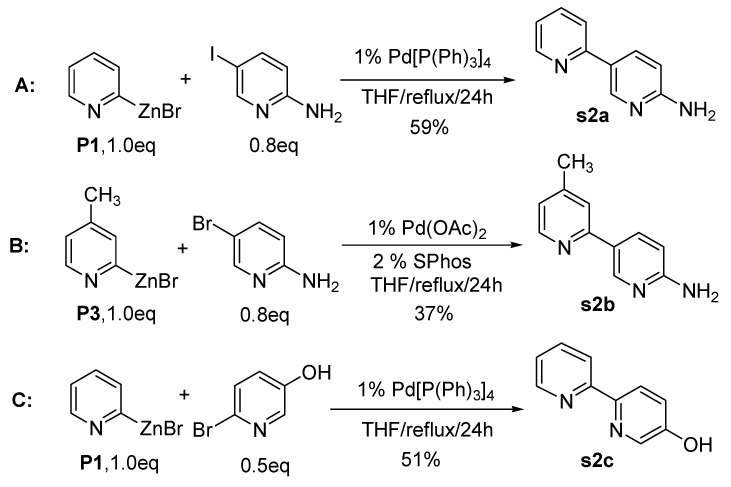
Preparation of amino and hydroxyl bipyridines.

Interestingly, unsymmetrical aminobipyridines were produced from the coupling reactions of 2-pyridylzinc bromides with halogenated aminopyridines under the conditions used above. As shown in Scheme 2, 2-amino-5-iodopyridine reacted with **P1** to afford 2,3-bipyridine (**s2a**) in 59% isolated yield in the presence of 1 mol% of Pd[P(Ph)_3_]_4 _catalyst (route A, Scheme 2). However, in the case of 2-amino-5-bromopyridine, the Pd(II)-catalyst was more efficient for the coupling reaction and the reaction proceeded smoothly to give 2,3-bipyridine (**s2b**, route **B**, Scheme 2). It is of interest that the bipyridylamines can be used as intermediates for the synthesis of highly functionalized molecules after transformation of the amino group to a halogen [70]. 

Treatment of 2-pyridylzinc bromide (**P1**) with a halopyridine bearing a hydroxyl group provided another functionalized bipyridine. Interestingly, the relatively reactive bromopyridyl alcohol, 2-bromo-5-hydroxypyridine, was coupled with **P1** using Pd(0)-catalyst. The hydroxyl group on 2,2’-bipyridine can also be converted to halogen to make halobipyridines using several different methods [71].

**Table 9:** Coupling reactions with haloaromatic alcohols.

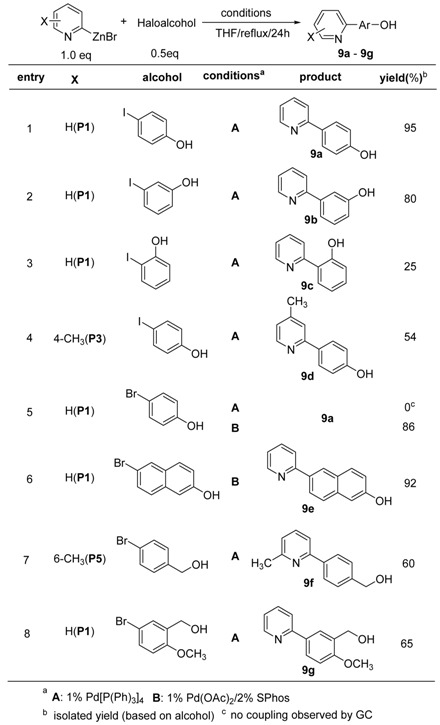

It has also been found that Rieke zinc in the presence of certain additives exhibits a very high reactivity towards 3-bromopyridine. The corresponding 3-pyridylzinc bromide was easily prepared by the direct insertion of active zinc and the resulting 3-pyridylzinc bromide was successfully applied to the cross-coupling reaction with a variety of electrophiles under mild conditions.

The first attempt to synthesize 3-pyridylzinc bromide from the direct reaction of active zinc and 3-bromopyridine in THF at either rt or refluxing temperature resulted in low conversion to the desired organozinc reagent. Almost the same result was obtained after an extended reaction time (reflux/24h). However, a dramatic improvement in the oxidative addition of active zinc has been achieved by adding 10–20 mol% of lithium chloride to the reaction mixture. Even though the role of lithium chloride has not been totally explained, more than 99% conversion of 3-bromopyridine to 3-pyridylzinc bromide was obtained in 2h at refluxing temperature in THF. As was pointed out in 1989, the rate limiting step in the oxidative addition is electron transfer [72]. Accordingly, this process will be accelerated by the presence of alkali salts which are generated in the reduction process of forming the active metals or additional salts can be added to the reaction mixture [61].

In order to confirm the formation of 3-pyridylzinc bromide, the resulting organozinc reagent was first treated with iodine, affording 90% 3-iodopyridine and 3% pyridine. The resulting 3-pyridylzinc bromide (**P7**) was added to a variety of different electrophiles to give the corresponding coupling products in moderate to good yields. The results are summarized in Table 10. Palladium catalyzed cross-coupling reactions with aryl iodides (**a** ~ **c**, Table 10) were completed in 1h at rt to give 3-pyridylbenzene derivatives **10a**, **10b**, and **10c** in good yields (entries 1 ~ 3, Table 10). A longer reaction time was required with aryl iodides **d** and **e**, bearing a substitutent in the 2-position (entries 4 and 5, Table 10). This is probably due to steric hindrance. Even though a low yield was obtained from 2,6-dibromopyridine (**j**), the coupling product **10j** bearing a bromine atom can serve as a valuable intermediate for the preparation of a variety of materials. Interestingly, it was also possible to obtain an aromatic ketone **10k** in moderate yield from the reaction of **P7** with benzoic acid anhydride in the presence of Pd(0) catalyst. 

**Table 10:** Pd-Catalyzed coupling reactions of 3-pyridylzinc bromide (**P7**).

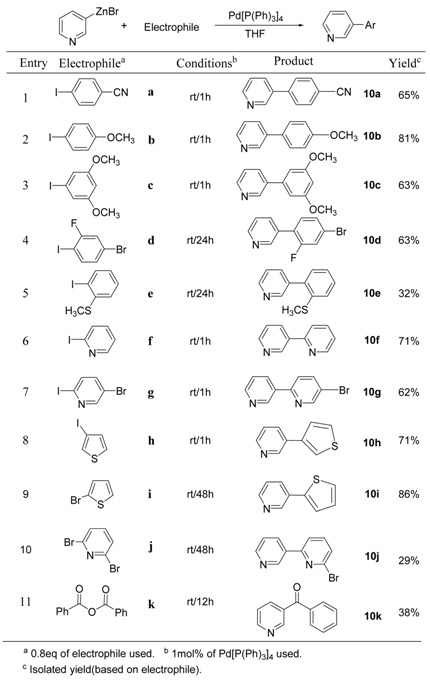

To expand the applications of 3-pyridylzinc bromide, several other copper-catalyzed coupling reactions were also investigated and the results are summarized in Table 11. S_N_2’-type reactions have been tried with allyl halides affording the resulting products (**11a** and **11b**, Table 11) in moderate to good yields. In the presence of TMSCl, silyl enol ether **11c** (Table 2) was obtained from the conjugate addition intermediate. Like other general organozinc reagents, 3-pyridylzinc bromide (**P7**) was successfully used for the copper-catalyzed synthesis of ketone compounds.

**Table 11:** Copper-catalyzed coupling reaction of 3-pyridylzinc bromide.

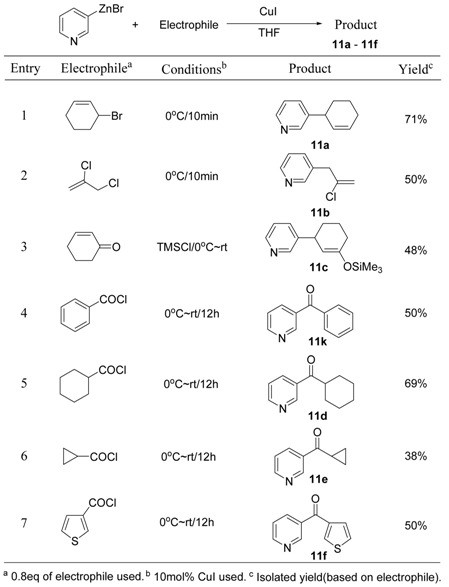

This study was expanded to several analogues of 3-bromopyridine. As described in Table 12, 3-bromoquinoline (**Q1**) and 3-bromoisoquinoline (**Q2**) were treated with active zinc along with 20 mol% of lithium chloride. It was found that the oxidative addition of active zinc was completed in 2h at refluxing temperature to give the corresponding organozinc reagents. The subsequent coupling reactions of **Q1** were performed with aryl iodide (entry 1, Table 12) and heteroaryl iodides (entries 2 and 3, Table 12) in the presence of palladium catalyst affording the corresponding products (**12a** ~ **12c**) in moderate to good isolated yields. 

**Table 12:** Preparation of quinoline and isoquinoline derivatives via heteroarylzinc reagent.

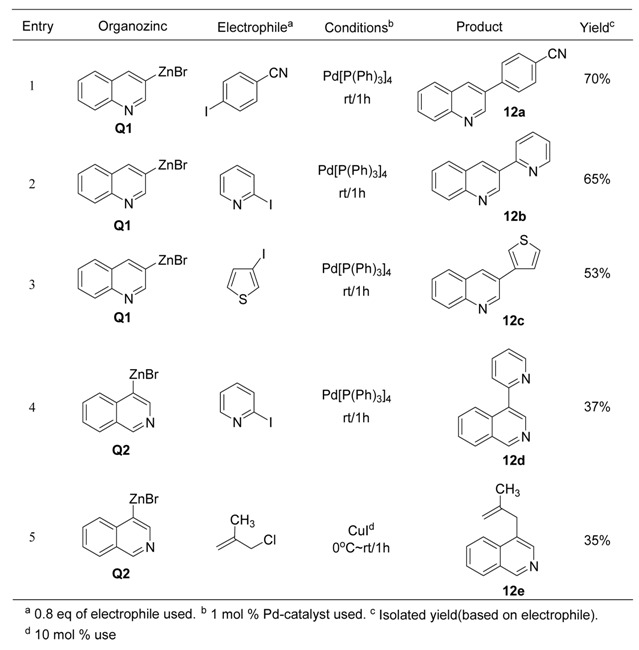

Along with the successful results from the coupling reaction of 2-pyridylzinc bromides with aromatic haloamines and alcohols, the readily available 3-pyridylzinc bromide was easily coupled with haloaromatic compounds bearing relatively acidic protons under mild conditions affording the corresponding cross-coupling products in the moderate to excellent yields. Of primary interest, the general procedure for the transition metal-catalyzed cross-coupling reactions of 3-pyridylzinc bromides with haloanilines and halophenols providing 3-(aminophenyl)pyridines and 3-(hydroxyl-phenyl) pyridines were reported. The results also include the preparation of quinoline and isoquinoline derivatives as well as other pyridine derivatives.

The first approach included the reaction of 3-pyridylzinc bromide (**P7**) with 4-iodoaniline in the presence of 1% of Pd(OAc)_2_ along with 2% of SPhos in THF (enty1, Table 13). Even though a little longer reaction time was required, the coupling product **13a** was also obtained in good yield from the reaction with 4-bromoaniline under the same conditions (entry 2, Table 13). Interestingly, trace amounts of coupling product was detected by GC from the reaction with 4-iodophenol using the same conditions (entry 3, Table 13). A protected bromophenol gave rise to the coupling product **13b** in moderate yield under the same conditions (entry 4, Table 13). As described in the previous report, the critical role of an extra ligand (SPhos) for the completion of the coupling reaction was observed. Trace amount of product formation was detected in the absence of SPhos (entry 5, Table 13). It was also found that a Pd(0)-catalyst was not effective for the coupling with a protected bromophenol (entry 6, Table 13).

**Table 13:** Preliminary test for the coupling reaction.

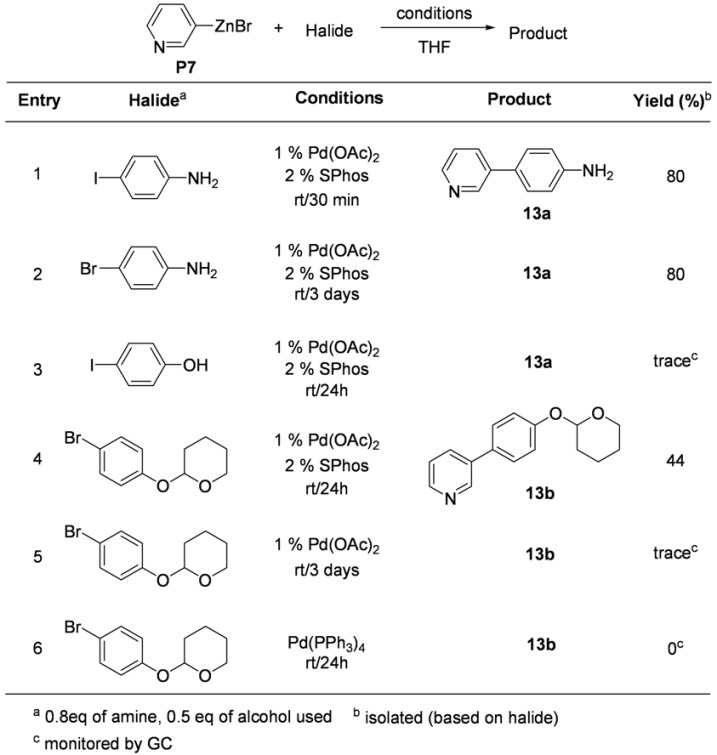

Again, extra ligand-free coupling reactions were also successfully performed in the case of 3-pyridylzinc with iodoanilines. The results are summarized in Table 14. No significant effect of the presence of acidic protons was observed in the Pd(0)-catalyzed coupling reactions.

The coupling reactions were easily accomplished by the addition of 3-pyridylzinc bromide into the mixture of haloaniline and Pd(0)-catalyst in THF. The organozinc solution was added into the reaction flask in one portion via a syringe at room temperature. The lack of a large heat of reaction should be a useful feature for large scale synthesis. For the sterically hindered 4-methyl-3-pyridylzinc bromide (**P8**), slightly more severe conditions (refluxing for 6 h) were required to complete the coupling. Unfortunately, no satisfactory coupling reaction occurred with 2-methoxy-5-pyridylzinc bromide (**P13**) using the Pd(0)-catalyst (entry 9, Table 14). From the results described above, it can be concluded that Pd(0)-catalyzed coupling reactions of 3-pyridylzinc bromides work effectively with iodoanilines under mild conditions. 

**Table 14:** Preparation of aminophenylpyridines.

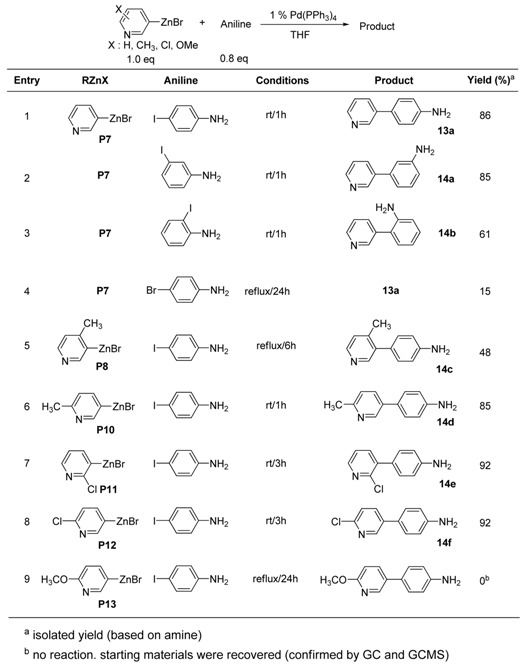

Another interesting reaction of 3-pyridylzinc bromide would be the coupling reaction with phenols, which also have an even more acidic proton [69]. The coupling reactions with iodophenols were carried out using Pd(0)-catalyst. As shown in Table 15, 2.0 equivalent of organozinc reagent was reacted with halophenols at refluxing temperature in the presence of 1 mol% of Pd(PPh_3_)_4_ in THF. In the case of **P7**, even though the coupling reaction with iodophenol worked fairly at rt, increasing the reaction temperature worked more effectively to complete the coupling reaction. Unlike the reaction with 4-bromoaniline, treatment of **P7** with 4-bromophenol gave rise to the product **15a** in moderate yield (entry 3, Table 15). 

**Table 15:** Preparation of hydroxyphenylpyridines.

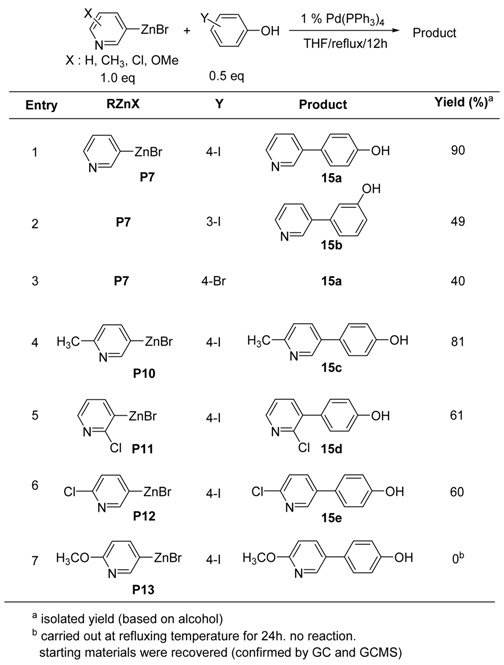

With the successful results described above, several analogues of 3-bromopyridine was also tried. The results are described in Table 16. Quinolinylzinc bromide (**Q1**) and isoquinolinylzinc bromide (**Q2**) were easily prepared and then the subsequent coupling reactions with 4-iodoaniline and 4-iodophenol afforded the corresponding products, **16a** and **16b**.

**Table 16:** Preparation of quinoline and isoquinoline derivatives.

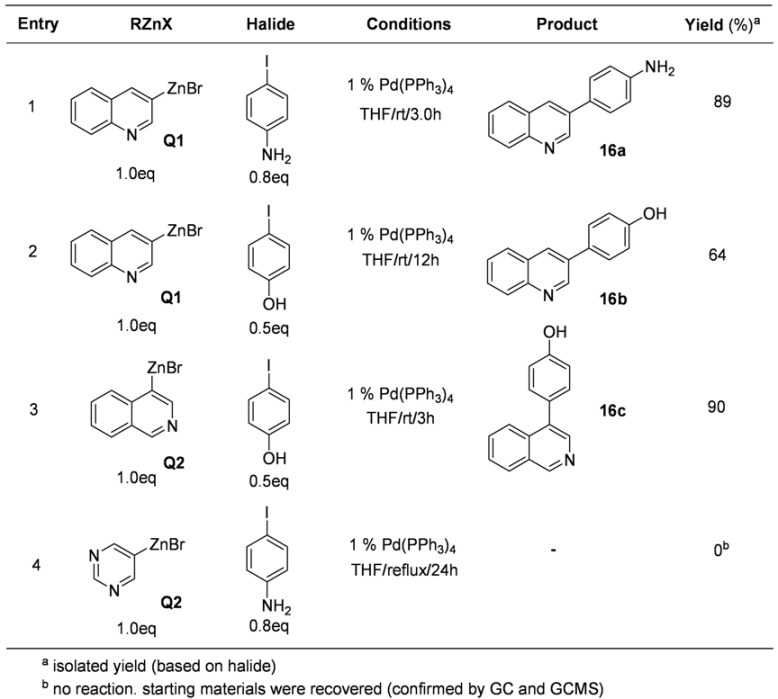

**Scheme 3:**
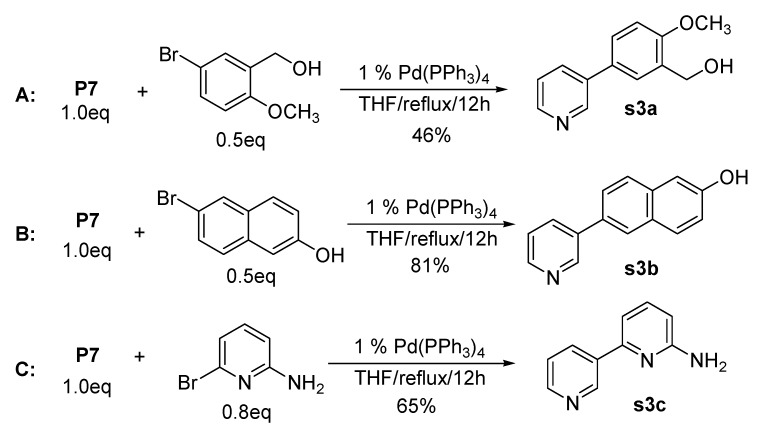
Expanded examples of coupling reactions.

Further application of this practical synthetic approach has been performed by the coupling reaction with different types of alcohols. 2-Methoxy-5-bromobenzyl alcohol and 6-bromo-2-naphthol were nicely coupled with **P7** under the reaction conditions given in Scheme 3. Interestingly, unsymmetrical amino-bipyridines were produced from the coupling reactions of **P7** with 2-amino-6-bromopyridine (route C, Scheme 3). It is of interest that the resulting product (**s3c**) can be utilized for further applications after transformation of the amino group to a halogen [70].

### 2.2. Heteroarylmanganese reagents

In spite of the importance of π-deficient heteroaryl manganese halides, few results have been reported about the preparation and application of functionalized heteroaromatic manganese reagents [73,74]. In general, heteroaryl manganese halides are easily prepared by the reaction of highly active manganese (Rieke manganese, Mn*) with various heteroaryl halides under mild reaction conditions. Significantly, cross-coupling reaction of these reagents with acid chlorides was accomplished without using any transition metal catalyst.

**Table 17:** 3-Thienylmanganese bromides with acid chlorides.

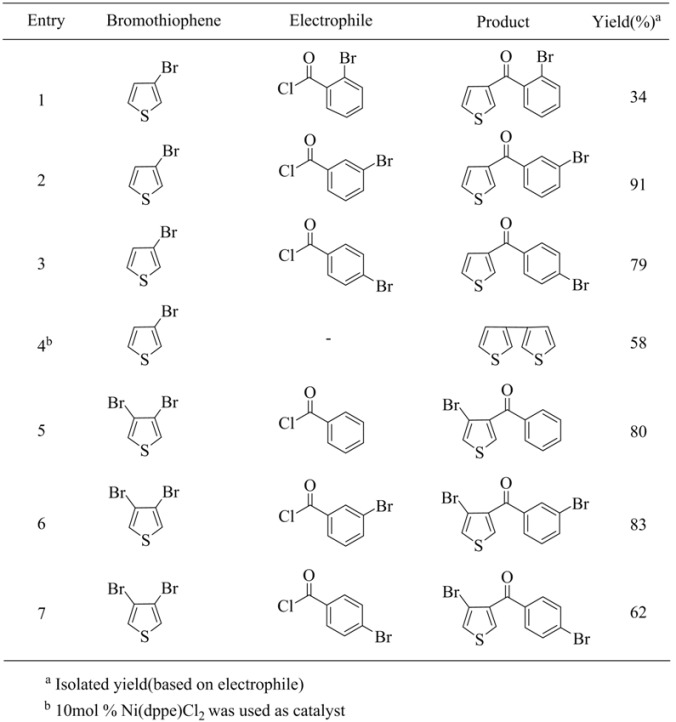

One of the interesting results was obtained using 3-thienylmanganese bromide, which was easily prepared from the reaction of 2 equiv of highly active manganese with 1 equiv of 3-bromothiophene. It is of interest that the mono-organomanganese bromide reagent is obtained from the reaction of highly active manganese with 3,4-dibromothiophene under the present conditions. For instance, 3-bromo-4-thienylmanganese bromide can be selectively produced as the major product from the reaction of 1 equiv of 3,4-dibromothiophene and 2 equiv of Mn*. According to the high resolution mass spectra of the products, the second bromine atom is still retained in the final products. In the presence of a catalytic amount of [1,2-bis(diphenylphosphine)ethane]nickel(II) chloride, homo-coupling product of 3-thienylmanganese bromide was obtained even using an electrophile such as aryl iodide (entry 4, Table 17). Once again, no transition metal catalysts are required to complete the cross-coupling reaction at room temperature. The result is summarized in Table 17.

Coupling reactions with aryl iodides were also investigated. These reactions were conducted in the presence of a catalytic amount of Pd[P(Ph)_3_]_4_ and resulted in the formation of coupling products in good yields under mild reaction conditions. Once again, selectively substituted 3-bromo-4-substituted thiophenes were easily obtained. The result is summarized in Table 18.

**Table 18:** Pd(0)-Catalyzed coupling reaction.^ a^

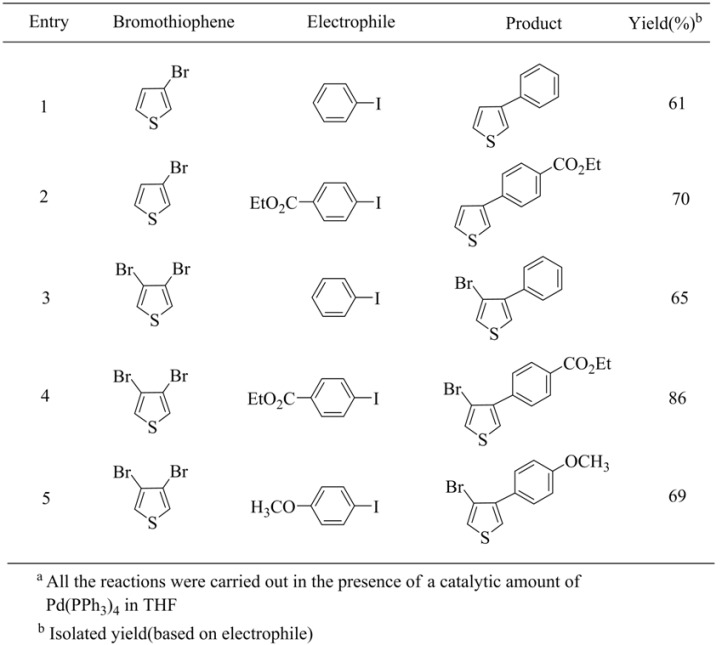

In addition, a variety of heteroarylmanganese reagents have been prepared using the procedure utilized in the preparation of thienylmanganese bromide. The resulting organomanganese reagents were successfully used in copper-catalyzed coupling reactions with acid chlorides (entries 1 ~ 9, Table 19) and Pd(0)-catalyzed coupling reactions with aryl iodides (Table 20). 

**Table 19:** Coupling reaction of heteroarylmanganese bromides.

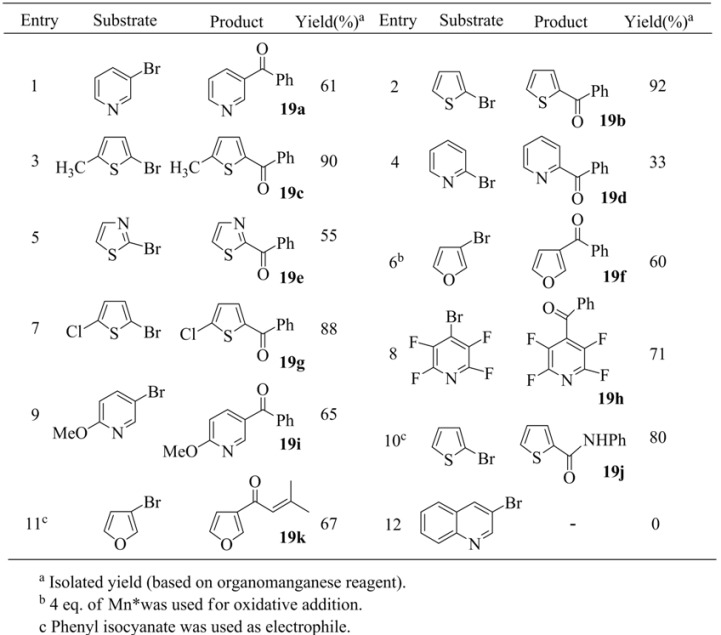

It was interestingly shown that furanic ketones or 3-acylfurans were easily obtained through the acylation of a 3-furyl manganese derivative. This is a significant result because these organometallic reagents are not available at rt due to their low stability [75,76]. However, the organomanganese reagents used in this study were readily prepared with highly active manganese and 3-bromofuran at room temperature. They have been used to prepare the derivative of natural furanic ketones (*perilla ketone*) [74]. Notably an alkene was easily coupled to give rise to the alkenylation product **2g** in moderate yield (entry 7, Table 20).

A number of transition metal catalyzed cross-coupling reactions between alkenyl halides and organometallics have been reported and used for the most useful methods for forming new carbon-carbon bonds between two C_sp_2-centers [78,79,80]. In these reactions, Pd(0), Ni(0), and Cu(I) have been widely used as catalyst [81,82,83,84,85]. However, it is often undesirable to use Pd- or Ni- complexes as catalyst for large-scale applications since they are expensive (Pd) or toxic (Pd, Ni). 

**Table 20:** Cross-coupling reactions of heteroarylmanganese reagents.^ a^

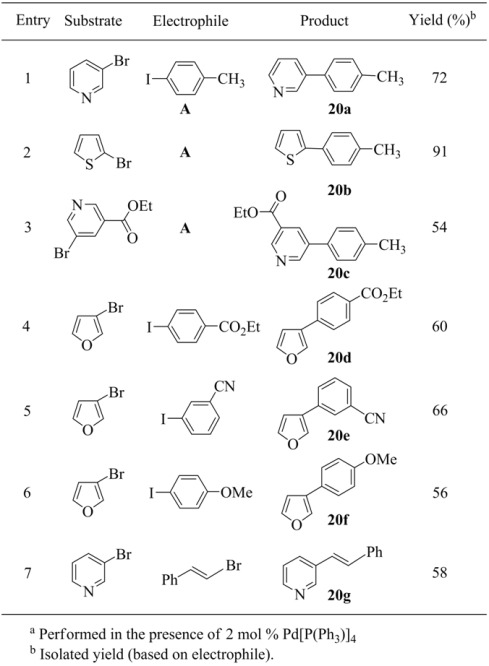

A previous report showed that alkenyl bromides were alkylated by Grignard reagents in the presence of non-toxic iron (III) acetylacetonate [86,87,88]. However, the preparative interest of this reaction is limited since a large excess of alkenyl bromide is required to obtain satisfactory yields of substituted olefins. Fe(III)-catalyzed cross-coupling reactions between alkyl- or aryl organometallic reagents and various alkenyl halides have also been reported offering an alternative to the Pd- and Ni-catalyzed procedure [89,90]. Cobalt is an alternative transition metal to be utilized for the cross-coupling alkenylation. Recently, it has been used as a catalyst for alkenylation of Grignard reagents [91,92].

Alkenylpyridines have received much attention as pharmacological interesting and synthetic intermediates. Accordingly, a large number of synthetic routes have been developed. For example, palladium-catalyzed cross-coupling reaction of organoborane compounds with various vinyl halides offers a potential method for the preparation of alkenyl substituted heteroaryl compounds [93,94]. Pd(0) was the most frequently used catalyst in these studies. 

**Table 21:** Co(II)-catalyzed alkenylation of pyridylmanganese reagents.

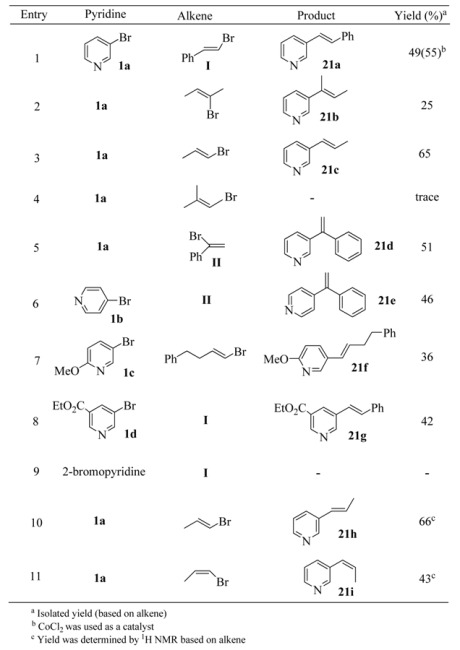

It was found that the best results were obtained using 10 mol% of cobalt catalyst [Co(acac)_2_ or CoCl_2_], 1.0eq of organomanganese reagent and 0.6eq of alkene. It has been found that the choice of addition sequence of the reagents is very important for optimizing the yield of cross-coupling products. When the organomanganese reagent was added first to a THF solution containing cobalt salts followed by addition of vinyl halides at the same temperature, no cross-coupling reaction was observed. Instead, all of the vinyl halides were recovered. The reason for this is not clear yet, but it can be concluded that organomanganese reagents may react with the cobalt catalyst first and then the resulting complex hinders the cross-coupling reaction. The results of Co(II)-catalyzed alkenylation of pyridylmanganese bromides with a variety of alkenyl bromides are summarized in Table 21.

## 3. Conclusions

In conclusion, a practical synthetic route for the preparation of 2-pyridyl and 3-pyridyl derivatives has been demonstrated. It has been accomplished by utilizing a simple coupling reaction of a stable 2-pyridylzinc bromides and 3-pyridylzinc bromides, which were prepared via the direct insertion of active zinc to the corresponding bromopyridines. The subsequent coupling reactions with a variety of different electrophiles have been carried out under mild conditions affording the coupling products. 

Highly active manganese prepared by the Rieke method has also shown unique chemical reactivity. Using active manganese, a variety of Grignard-type organomanganese reagents have been easily prepared by simple addition of corresponding heteroarylhalides to the highly active manganese. Subsequent coupling reactions of the resulting organomanganese reagents such as pyridylmanganese and thienylmanganese with several electrophiles have also been accomplished under mild conditions. 
